# Reachability Analysis Using Message Passing over Tree Decompositions

**DOI:** 10.1007/978-3-030-53288-8_30

**Published:** 2020-06-13

**Authors:** Sriram Sankaranarayanan

**Affiliations:** 8 Microsoft Research Lab, Redmond, WA USA; 9 University of Southern California, Los Angeles, CA USA; University of Colorado, Boulder, CO USA

## Abstract

In this paper, we study efficient approaches to reachability analysis for discrete-time nonlinear dynamical systems when the dependencies among the variables of the system have low treewidth. Reachability analysis over nonlinear dynamical systems asks if a given set of target states can be reached, starting from an initial set of states. This is solved by computing conservative over approximations of the reachable set using abstract domains to represent these approximations. However, most approaches must tradeoff the level of conservatism against the cost of performing analysis, especially when the number of system variables increases. This makes reachability analysis challenging for nonlinear systems with a large number of state variables. Our approach works by constructing a dependency graph among the variables of the system. The tree decomposition of this graph builds a tree wherein each node of the tree is labeled with subsets of the state variables of the system. Furthermore, the tree decomposition satisfies important structural properties. Using the tree decomposition, our approach abstracts a set of states of the high dimensional system into a tree of sets of lower dimensional projections of this state. We derive various properties of this abstract domain, including conditions under which the original high dimensional set can be fully recovered from its low dimensional projections. Next, we use ideas from message passing developed originally for belief propagation over Bayesian networks to perform reachability analysis over the full state space in an efficient manner. We illustrate our approach on some interesting nonlinear systems with low treewidth to demonstrate the advantages of our approach.

## Introduction

Reachability analysis asks whether a target set of states is reachable over a finite or infinite time horizon, starting from an initial set for a dynamical system. This problem is fundamental to the verification of systems, and is known to be challenging for a wide variety of models. This includes cyber-physical systems, physical and biological processes. In this paper, we study reachability analysis algorithms for nonlinear, discrete-time dynamical systems. The key challenge in analyzing such systems arises from the difficulty of representing the reachable sets of these systems. As a result, we resort to over-approximations of reachable sets using tractable set representations such as intervals [16], ellipsoids, polyhedra [19], and low degree semi-algebraic sets [2]. Whereas these representations are useful for reachability analysis, they also trade off the degree of over-approximation in representing various sets against the complexity of performing operations such as intersections, unions, projections and image computations over these sets. The theory of abstract interpretation allows us to design various abstract domains that serve as representations for sets of states in order explore these tradeoffs [17, 18, 34]. However, for nonlinear dynamical systems, these representations often become too conservative or too expensive as the number of state variables grow.

In this paper, we study reachability analysis using the idea of tree decompositions over the dependency graph of a dynamical system. Tree decompositions are a well-known idea from graph theory [37], used to study properties of various types of graphs. The treewidth of a graph is an intrinsic property of a graph that relates to how “far away” a given graph is from a tree. For instance, trees are defined to have a treewidth of 1. Many commonly occurring families of graphs such as *series-parallel graphs* have treewidth 2 and so on. Formally, a tree decomposition of a graph is a tree whose nodes are associated with subsets of vertices of the original graph along with some key conditions that will be described in Sect. 2. We use tree decompositions to build an abstract domain. The abstraction operation projects a set of states in the full system state space along each of the nodes of the tree, yielding various projections of this set. The concretization combines projections back into the high dimensional set. We study various properties of this abstract domain. First, we characterize abstract elements that can potentially be generated by projecting some concrete elements along the nodes of the tree (so called *canonical* elements, Definition 10). Next we characterize those sets which can be abstracted along the tree decomposition and reconstructed without any loss in information (tree decomposable sets, Definition 11). In this process, we also derive a *message passing* approach wherein nodes of the tree can exchange information to help refine sets of states in a sound manner. However, as we will demonstrate, the abstraction is “lossy” in general since projections of tree decomposable sets are not necessarily tree decomposable. We discuss some interesting ways in which precision can be regained by carefully analyzing this situation.

We combine these ideas together into an approach for reachability analysis of nonlinear systems using a grid domain that represents complex non convex sets as a union of fixed size cells using a gridding of the state-space. Although such a domain would be prohibitively expensive, we show that the tree decomposition abstract domain can drastically cut down on the complexity of computing reachable set overapproximations in this domain, yielding precise reachable set estimation for some nonlinear systems with low treewidth. We demonstrate our approach using a prototype implementation to show that for a restricted class of systems whose dependency graphs have low treewidth, our approach can be quite efficient and precise at the same time. Although some interesting systems have low treewidth property, it is easy to see that many systems will have treewidths that are too high for our approach. Our future work will consider how systems whose dependency graphs do not have sufficiently low treewidth can still be tackled in a conservative manner using some ideas from this paper.

### Related Work

As mentioned earlier, the concept of tree decompositions and treewidth originated in graph theory [37]. The concept of treewidth gained popularity when it was shown that many NP-complete problems on graphs such as graph coloring could be solved efficiently for graphs with small treewidths [5]. Courcelle showed that the problem of checking if a given graph satisfies a formula in the monadic second order logic of graphs can be solved in linear time on graphs with bounded treewidth [15]. Several NP-complete problems such as 3-coloring can be expressed in this logic. Tree decompositions are also used to solve inference problems over Bayesian networks leading to representations of the Bayesian networks such as junction trees that share many of the properties of a tree decomposition [29]. In fact, belief propagation over junction trees is performed by passing messages that marginalize the probability distributions at various nodes of the tree. This is analogous to the message passing approach described here.

Tree decomposition techniques have been applied to model checking problems over finite state systems. For instance, Obdržálek show that the $\mu$-calculus model checking problem can be solved in linear time in the size of a finite-state system whose graph has a bounded treewidth [35]. However, as Ferrara et al. point out, requiring the state graph of a system to have a bounded treewidth is often restrictive [24]. Instead, they study concurrent finite state systems wherein the communication graph has a bounded tree width. However, they conclude that while it is more reasonable to assume that the communication graph has a bounded tree width, it does not confer much advantages to verification problems. For instance, they show that the unrolling of these systems over time potentially results in unbounded treewidth. In this paper, we consider a different approach wherein we study the treewidth of dependency graphs of the system. We find that many systems have small treewidth and exploit this property. At the same time, we note that some of the benchmarks studied have “sparse” dependency graphs but treewidths that are too large for our approach.

Tree decomposition techniques have also been studied in static analysis of programs. The control and data flow graphs of structured programs without goto-statements or exceptional control flow are known to have small treewidth that can be exploited to perform compiler optimizations such as register allocation quite efficiently [38]. Chatterjee et al. have shown how to exploit small treewidth property of the control flow graphs of procedures in programs to perform interprocedural dataflow analysis by modeling the execution of programs with procedures as recursive state machines [11]. However, this approach seems restricted to control dominated properties such as sequence of function calls. In a followup work, they study control and data flow analysis problems for concurrent systems, wherein each component has constant treewidth [10]. In contrast, our approach studies dynamical system and consider tree decompositions of the data dependency graph.

The use of message passing in this paper closely resembles past work by Gulwani and Jojic [27]. Therein, a program verification problem involving the verification pre/post and intermediate assertions in a program is solved by passing messages that can propagate information between assertions along program paths in a randomized fashion. The approach is shown to be similar to loopy belief propagation used in Bayesian inference. The key differences are (a) we use data dependencies and tree decompositions rather than control flow paths to pass information along; and (b) we formally prove properties of the message passing algorithm.

Our approach is conceptually related to a well-known idea of speeding up static analysis of large programs using “packing” of program variables [4, 28]. This approach was used successfully in the Astreé static analyzer [3, 4, 21]. Therein, clusters of variables representing small sets of dependent local and global are extracted. The remaining program variables are abstracted away and the abstract interpretation process is carried out over just these variables. The usefulness of this approach has borne out in other abstract interpretation efforts, including Varvel [28]. The key idea in this paper can be seen as a formalization of the rather informal “clustering” approach using tree decompositions. We demonstrate theoretical properties as well as the ability to pass messages to improve the results of the abstract interpretation.

The use of the dependency graph structure to speed up reachability analysis approaches has been explored in the past for speeding up Hamilton-Jacobi-based approaches by Mo Chen et al. [12] as well as flowpipe based approaches by Xin Chen et al. [13]. Both approaches consider the directed dependency graph wherein $x_i$ is connected to $x_j$ if the former appears in the dynamical update equation of the latter variable. The approaches perform a strongly connected component (SCC) decomposition and analyze each SCC in a topological sorted order. However, this approach breaks as soon as the system has large SCCs, which is common. As a result, Xin Chen et al. show how SCCs can themselves be broken into numerous subsets at the cost of a more conservative solution. In contrast, the tree decomposition approach can be applied to exploit sparsity even when the entire dependency graph is a single SCC.

## Preliminaries

In this section, we will describe the system model under analysis, the dependency graph structure and the basics of tree decompositions. Let $X: \{x_1, \ldots , x_n\}$ be a set of *system variables* and $\mathbf {x}: X \mapsto \mathbb {R}$ represent a valuation to these system variables. Let *D* be the domain of all valuations of *X*, that describes the *state space* of the system. For convenience let $\mathbf {x}_i$ denote $\mathbf {x}(x_i)$. Also, let $W: \{w_1, \ldots , w_m\}$ represent disturbance variables and $\mathbf {w}: W \mapsto \mathbb {R}$ represent a vector of $m \ge 0$ external disturbance inputs that take values in some compact disturbance space $\mathcal {W}$.

### Definition 1 (Dynamical Model)

A model $\varPi$ is a tuple $\left\langle X, W, D, \mathcal {W}, f,\right. \left. X_0, U\right\rangle$, wherein $X, W, D, \mathcal {W}$ are as defined above, *f* is an arithmetic expression over variables in *X*, *W* describing the dynamics, $X_0$ is a set of possible initial valuations (states) and *U* is a designated set of unsafe states.

The dynamics are given by $\mathbf {x}(t+1) = \mathsf {eval}(f, \mathbf {x}, \mathbf {w})$, wherein $\mathsf {eval}$ evaluates a given an expression *f*, a set of valuations to the system variables $\mathbf {x}\in D$ and disturbances $\mathbf {w}\in \mathcal {W}$, and returns a new set of valuations for each variable in *X*, denoted by $\mathbf {x}(t+1)$.

For simplicity, we write $f(\mathbf {x},\mathbf {w})$ to denote $\mathsf {eval}(f, \mathbf {x},\mathbf {w})$ for a function expression *f*. A state of the system is a valuation $\mathbf {x}: X \mapsto \mathbb {R}$ such that $\mathbf {x}\in D$. Given a finite sequence of disturbance inputs $\mathbf {w}(0), \ldots , \mathbf {w}(T)$, for some $T \ge 0$ and $\mathbf {w}(i) \in \mathcal {W}$ for all $i \in [0,T]$, an execution of the system is a sequence of states $\mathbf {x}(0), \ldots , \mathbf {x}(T+1)$, such that $\mathbf {x}(0) \in X_0$, $\mathbf {x}(t) \in D$ for $t \in [0,T+1]$ and $\mathbf {x}(t+1) = f(\mathbf {x}(t), \mathbf {w}(t))$ for all $t \in [0, T]$. According to these semantics, the system may fail to have an execution for a given disturbance sequence $\mathbf {w}(t),\ t \in [0, T]$ and initial state $\mathbf {x}(0)$ if for some state $\mathbf {x}(t)$, we have $f(\mathbf {x}(t), \mathbf {w}(t)) \not \in D$.

A state $\mathbf {x}(t)$ is reachable (at time *t*) if there is an execution of the form $\mathbf {x}(0), \ldots , \mathbf {x}(t)$, satisfying the constraints above. We say that the unsafe state *U* is reachable iff some state $\mathbf {x}\in U$ is reachable. Furthermore, we say that *U* is reachable within a finite time horizon *T*, iff some state $\mathbf {x}\in U$ is reachable at time $t \in [0, T]$.

### Example 1

Consider a nonlinear example of a dynamical model $\varPi$ with state space $\mathbf {x}: (x_1, x_2, x_3)$ and $\mathbf {w}: (w_1)$. The dynamics can be written as parallel assignments to the state variables:

$$\begin{array}{c} x_1\ :=\ x_1 + 0.25 x_2 - 0.05 x_1 sin(x_2),\ x_2\ :=\ x_2 + w_1, \ x_3 \ := x_3 - 0.2 x_3 x_2\,,\\ \end{array}$$

The assignments are all evaluated in parallel to update the current state $\mathbf {x}(t)$ to a new state $\mathbf {x}(t+1)$. The domain *D* is $x_i \in [-3,3]$ for $i = 1,2,3$ and the disturbance $w_1 \in [-0.1, 0.1]$. The initial set $X_0$ is $x_1 \in [-0.2,0.2]\ \wedge \ x_2 \in [-0.3, 0] \ \wedge \ x_3 \in [0, 0.4]$.

We will now define the dependency (hyper)graph of the system $\varPi$. For convenience, we write the update function (expression) *f* of a system $\varPi$ in terms of individual updates $(f_1, \ldots , f_n)$, wherein $x_j' = f_j(\mathbf {x}, \mathbf {w})$. We say that system variable $x_i$ (or disturbance variable $w_j$) is a *proper input* to the expression $f_k$ if $x_i$ (or $w_j$) occurs as a subterm in $f_k$. Let $\mathsf {inps}(f_k)$ denote the set of all proper input variables to the function (expression) $f_k$.

As an example, consider $$X = \{x_1, \ldots , x_4 \}$$ and $$W = \{w_1, w_2\}$$ and the expression $$f: x_1 x_4 - w_1 $$. The proper inputs to *f* are $$\{ x_1, x_4, w_1\}$$. We exclude cases such as $$g: \frac{\sin ^2(x_1)\,+\,\cos ^2(x_1)}{\sin ^2(x_2)\,+\,\cos ^2(x_2)}$$ that has $$\{x_1, x_2\}$$ as proper inputs. However a simplification using elementary trigonometric rules can eliminate them. We will assume that all expressions are simplified to involve the least number of variables.

### Definition 2 (Dependency Hypergraph)

A dependency hypergraph of a system $\varPi$ has vertices $V:\ X \cup W$, given by the union of the system and disturbance variables with hyperedge set $E \subseteq 2^{V}$ given by $E = \{ e_1, \ldots , e_n \}$, wherein for each update $x_k := f_k(\mathbf {x}, \mathbf {w})$ ($k = 1, \ldots , n$), we have the hyperedge $e_k : \{x_k \} \cup \mathsf {inps}(f_k)$. In other words, each update $x_k := f_k(\mathbf {x}, \mathbf {w})$ yields an edge that includes $x_k$ along with all the system/disturbance variables that are proper inputs to $f_k$.

### Example 2

The dependency hypergraph for the system from Example 1 has the vertices $V: \{ x_1, x_2, x_3, w_1\}$ and the edges $\{ e_1: \{ x_1, x_2 \},\ e_2:\ \{ x_2, w_1 \}$ and $e_3:\ \{ x_2, x_3\}\}$.

### Tree Decomposition

We will now discuss tree decompositions and the associated concept of treewidth of a hypergraph *G* : (*V*, *E*). The tree decomposition will be applied to the dependency hypergraphs (Definition 2) for systems $\varPi$ (Definition 1).

#### Definition 3 (Tree Decomposition and Treewidth)

Given a hypergraph *G* : (*V*, *E*), a tree decomposition is a tree *T* : (*N*, *C*) and a mapping $\textsc {verts}: N \mapsto 2^V$, wherein *N* is the set of tree nodes, *C* is the set of tree edges and $\textsc {verts}(\cdot )$ associates each node $u \in N$ with a set of graph vertices $\textsc {verts}(n) \subseteq V$. The tree decomposition satisfies the following conditions:

1. For vertex $v \in V$ there exists (at least one) $n \in N$ such that $v \in \textsc {verts}(n)$.
2. For each hyperedge $e \in E$ there exists (at least one) $n \in N$: $e \subseteq \textsc {verts}(n)$.
3. For each vertex *v*, for any two nodes $n_1, n_2$ such that $v \in \textsc {verts}(n_1)$ and $v \in \textsc {verts}(n_2)$, then $v \in \textsc {verts}(n)$ for each node *n* along the unique path between $n_1$ and $n_2$ in the tree. Stated another way, the subset of nodes $N_v: \{ n \in N\ |\ v \in \textsc {verts}(n) \}$ induces a subtree of *T* (denoted $T_v$).

The width of a tree decomposition is given by $\max \{ |\textsc {verts}(n)|\ |\ n \in N \} -1$. In other words, we find the node *n* in the tree whose associated set of vertices has the largest cardinality. We subtract one from this maximal cardinality to obtain the treewidth. A tree decomposition is optimal for a graph *G* if no other tree decomposition exists with a strictly smaller width. The treewidth of a hypergraph *G* is given by width of an optimal tree decomposition.

It is easy to show that if the graph *G* is a tree, it has treewidth 1. Likewise, a cycle has tree width 2.

#### Example 3

The tree decomposition of the hypergraph *G* from Example 2 has three nodes $\{ n_1, n_2, n_3 \}$ with edges $(n_1, n_2)$ and $(n_2, n_3)$. The nodes along with the associated vertex sets are as follows: 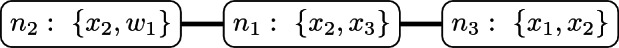


Although the tree decomposition is not a rooted tree, we often designate an arbitrary node $r \in N$ as the root node, and consider the tree *T* as a rooted tree with root *r*.

**Finding a Tree Decomposition:** Interestingly, the problem of finding the treewidth of a graph is itself a NP-hard problem. However, many practical approaches exist for graphs with small treewidths. For instance, Bodlaender presents an algorithm that runs in time $O(k^{O(k^3)})$ to construct a tree decomposition of width at most *k* or conclude that the treewidth of the graph is at least $k+1$ [6]. Such an approach can be quite useful if a given graph is suspected to have a small tree width in the first place. Besides this, many efficient algorithms exist to approximate the treewidth of a graph to some constant factor. A detailed survey of these results is available elsewhere [7, 8]. Open-source packages such as HTD can compute treewidth for graphs with thousands of nodes [1]. Finally, we note that if a tree decomposition of width *k* can be found, then one can be found with at most |*V*| nodes.

#### Lemma 1

Let *T* be a tree decomposition for a (multi)graph *G* with vertices *V* and treewidth *k*. There exists a tree decomposition $\hat{T}$ of *G* with the same treewidth *k*, and at most |*V*| nodes.

A proof is provided in the extended version of the paper.

## Abstract Domains Using Tree Decompositions

In this section, we will define abstract domains using tree decompositions of the dependency hypergraph of the system under analysis. Let $\varPi$ be a transition system over system variables *X*. The concrete states are given by $\mathbf {x}\in D$, wherein $\mathbf {x}: X \mapsto \mathbb {R}$ maps each state variable $x_j \in X$ to its value $\mathbf {x}(x_j)$ (denoted $\mathbf {x}_j$).

### Definition 4 (Projections)

The projection of a state $\mathbf {x}$ to a subset of state variables $J \subseteq X$, denoted as $\mathsf {proj}(\mathbf {x}, J)$, is a valuation $\hat{\mathbf {x}}: J \mapsto \mathbb {R}$ such that $\hat{\mathbf {x}}(x_i) = \mathbf {x}(x_i)$ for all $x_i \in J$. For a set of states $S \subseteq D$ and a subset of state variables $J \subseteq X$, we denote the projection of *S* along (the dimensions of) *J* as $\mathsf {proj}(S, J): \{ \mathsf {proj}(\mathbf {x}, J)\ |\ \mathbf {x}\in S\}$.

### Definition 5 (Extensions)

Let *R* be a set of states involving just the variables in the set $J_1 \subseteq X$, i.e, $R \subseteq \mathsf {proj}(D, J_1)$. We define the *extension* of *R* into a set of variables $J_2 \supseteq J_1$ as $\mathsf {ext}_{J_2}(R):\ \{ \mathbf {x}\in \mathsf {proj}(D, J_2)\ |\ \mathsf {proj}(\mathbf {x}, J_1) \in R \}$.

In other words, the extension of a set embeds each element in the larger dimensional space defined by $J_2$ allowing “all possible values” for the dimensions in $J_2 \setminus J_1$.

We will use the notation $\mathsf {ext}(S)$ to denote the set $\mathsf {ext}_{X}(S)$, i.e, its extension to the entire set of state variables *X*. For a state $\mathbf {x}_S$, we will use $\mathsf {ext}(\mathbf {x}_S)$ denote $\mathsf {ext}(\{\mathbf {x}_S\})$.

### Definition 6 (Product (Join) of Sets)

Let $R_1 \subseteq \mathsf {proj}(D, J_1)$ and $R_2 \subseteq \mathsf {proj}(D, J_2)$. We define $R_1 \otimes R_2:\ \{ \mathbf {x}: J_1 \cup J_2 \mapsto \mathbb {R}\ |\ \mathsf {proj}(\mathbf {x}, J_1) \in R_1 \ \text {and}\ \mathsf {proj}(\mathbf {x}, J_2) \in R_2 \}$.

Let *T* : (*N*, *C*) be a tree decomposition of the dependency hypergraph of the system. Recall that for each node $n \in N$ we associate a set of system/disturbance variables denoted by $\textsc {verts}(n)$. Let $\textsc {verts}_X(n)$ denote the set of system variables: $\textsc {verts}(n) \cap X$. We say that an update function $x_k := f_k(\mathbf {x}, \mathbf {w})$ is associated with a node *n* in the tree iff $\{ x_k \} \cup \mathsf {inps}(f_k) \subseteq \textsc {verts}(n)$.

### Lemma 2

For every system variable $x_k$, its update $x_k \ :=\ f_k(\mathbf {x}, \mathbf {w})$ is associated with at least one node $n \in N$.

### Proof

This follows from those of a tree decomposition that states that every hyperedge in the dependency hypergraph must belong to $\textsc {verts}(n)$ for at least one node $n \in N$.

### Abstraction and Concretization

We consider subsets of the concrete states for the system $\varPi$, i.e, the set $2^D$, ordered by set inclusion as our *concrete domain*. Given a tree decomposition, *T*, we define an abstract domain through projection of a concrete set along $\textsc {verts}(n)$ for each node *n* of *T*.

#### Definition 7 (Abstract Domain)

Each element *s* of the abstract domain $\mathbb {A}_T$ is a mapping that associates each node $n \in N$ with a set $s(n) \subseteq \mathsf {proj}(D, \textsc {verts}_X(n))$.

For $s_1, s_2 \in \mathbb {A}_T$, $s_1 \sqsubseteq s_2$ iff $s_1(n) \subseteq s_2(n)$ for each $n \in N$.

We will use the notation $\mathsf {proj}(S, n)$ for a node $n \in N$ to denote $\mathsf {proj}(S, \textsc {verts}_X(n))$.

#### Definition 8 (Abstraction Map)

Given a tree decomposition *T*, the abstraction map $\alpha _T$ takes a set of states $S \subseteq D$ and produces a mapping that associates tree node $n \in N$ to a projection of *S* along the variables $\textsc {verts}_X(n)$. Formally,

$$\begin{aligned} \alpha _T(S) :\ \lambda n:N.\ \mathsf {proj}(S, n) \,. \end{aligned}$$

Thus, an abstract state *s* is a map that associates each node *n* of the tree to a set $s(n) \subseteq D_n$. We now define the concretization map $\gamma _T$.

#### Definition 9 (Concretization Map)

The concretization $\gamma _T(s)$ of an abstract state is defined as $\gamma _T(s):\ \bigcap _{n\in N} \mathsf {ext}(s(n))$. In other words, we take *s*(*n*) for every node $n \in N$, extend it to the full dimensional space of all system variables and intersect the result over all nodes $n \in N$.

#### Example 4

Consider a simple tree decomposition *T* with 2 nodes $n_1, n_2$ and a single edge $(n_1, n_2)$. Let $\textsc {verts}(n_1): \{ x_1, x_2\}$ and $\textsc {verts}(n_2): \{x_2, x_3\}$. Let the domain *D* be the set $\mathbf {x}_i \in \{1, 2, 3\}$ for $i = 1,2,3$. We use the notation  to denote a state $\mathbf {x}$ that maps $x_1$ to the value $v_1$, $x_2$ to the value $v_2$ and so on.

Now consider the set . We have that $s: \alpha (S)$ is the mapping that projects *S* onto the dimensions $(x_1, x_2)$ for node $n_1$ and $(x_2, x_3)$ for node $n_2$:Likewise, we verify that the concretization map $\gamma (s)$ will yields us:


For convenience, if the tree *T* is clear from the context, we will drop the subscripts to simply write $\alpha$ and $\gamma$ for the abstraction and concretization map, respectively.

#### Theorem 1

For any tree decomposition *T*, the maps $\alpha$ and $\gamma$ form a Galois connection. I.e, for all $S \subseteq D$ and $s \in \mathbb {A}_T$: $\alpha (S) \sqsubseteq s\ \text{ iff }\ S \subseteq \gamma (s)$.

#### Proof

Let *S*, *s* be such that $\alpha (S) \sqsubseteq s$. Therefore, $\mathsf {proj}(S, n) \subseteq s(n)\ \forall n \in N$ by the definition of $\sqsubseteq$. Pick any, $\mathbf {x}\in S$. First, $\mathsf {proj}(\mathbf {x}, n) \in \mathsf {proj}(S, n)$ and therefore, $\mathsf {proj}(\mathbf {x}, n) \in s(n)$ for all $n \in N$. Thus, $\mathbf {x}\in \mathsf {ext}(s(n))$ for each node $n \in N$. Therefore, $\mathbf {x}\in \bigcap _{n \in N} \mathsf {ext}(s(n))$, and hence, $\mathbf {x}\in \gamma (s)$, by defn. of $\gamma$. Therefore, $S \subseteq \gamma (s)$.

Conversely, assume $S \subseteq \gamma (s)$. Since $\gamma (s) = \bigcap _{n \in N} \mathsf {ext}(s(n))$ (from Definition 9). Therefore, $S \subseteq \mathsf {ext}(s(n))$ forall $n \in N$. Therefore, for all $\mathbf {x}\in S$, $\mathsf {proj}(\mathbf {x}, n) \in s(n)$. Therefore, $\mathsf {proj}(S, n) \subseteq s(n)$ for every $n \in N$. Finally, this yields $\alpha (S) \sqsubseteq s$.

The meet operation is defined as $s_1 \sqcap s_2:\ \lambda n.\ s_1(n) \cap s_2(n)$, and likewise, the join is defined as $s_1 \sqcup s_2:\ \lambda n.\ s_1(n) \cup s_2(n)$. We recall two key facts that follow from Galois connection between $\alpha$ and $\gamma$.

1. For any set $S \subseteq D$, we have $S \subseteq \gamma (\alpha (S))$. Abstracting a concrete set and concretizing it back again “loses information”. To see why, we start from $\alpha (S) \sqsubseteq \alpha (S)$ and apply the Galois connection to derive $S \subseteq \gamma (\alpha (S))$.
2. Likewise, for any abstract domain object $s \in \mathbb {A}$, we have $\alpha (\gamma (s)) \sqsubseteq s$. I.e, for any element *s*, taking its concretization and abstracting it “gains information”. To prove this, we start from $\gamma (s) \subseteq \gamma (s)$ and conclude that $\alpha (\gamma (s)) \sqsubseteq s$.

#### Example 5

Returning back to Example 4, now consider the setIts abstraction $\hat{s}: \alpha (\hat{S})$ is given by the mapping:We note that $\gamma (\hat{s})$ is the set: . Thus $\hat{S} \subseteq \gamma (\hat{s})$. Notice that  and  are part of $\gamma (\hat{s})$ but not the original set $\hat{S}$. Similarly, consider the abstract element $s_1$: . We note that  and therefore $\alpha (\gamma (s_1))$ yields the abstract element $s_2 \sqsubseteq s_1$: .

### Canonical Elements and Message Passing

In the tree decomposition, various nodes share information about the subsets of vertices associated with each node. Since the subsets have elements in common, it is possible that a node $n_1$ has information about a variable $x_2$ that is also present in some other node $n_2$ of the tree. We will now see how to take an abstract element *s* and refine each *s*(*n*) by exchanging information between nodes in a systematic manner.

For each edge $(n_1, n_2) \in C$ of the tree, define the set of variables in common as $\textsc {CV}(n_1, n_2){:}\ \textsc {verts}(n_1) \cap \textsc {verts}(n_2)$ and $\textsc {CV}_X(n_1, n_2){:}\ \textsc {verts}_X(n_1) \cap \textsc {verts}_X(n_2)$.

#### Definition 10 (Canonical Elements)

An abstract element *s* is said to be *canonical* if and only if for each edge $(n_1, n_2) \in C$ in the tree:

$$\begin{aligned} \mathsf {proj}(s(n_1), \textsc {CV}_X(n_1, n_2)) = \mathsf {proj}(s(n_2), \textsc {CV}_X(n_1, n_2)) \,. \end{aligned}$$

In other words, if we took the common variables $\textsc {verts}_X(n_1) \cap \textsc {verts}_X(n_2)$, the set $s(n_1)$ projected along these common variables is equal to the projection of $s(n_2)$ along the common variables.

#### Example 6

Consider the abstract element $s_1$ from Example 5: .  is the set  whereas $\mathsf {proj}(s_1(n_2), \textsc {CV}(n_1, n_2))$ is simply . Therefore, $s_1$ fails to be canonical.

The key theorem of tree decomposition is that a canonical element in the abstract domain can be seen as the projection of a concrete set *S* along $\textsc {verts}_X(n)$ for each node *n* of the tree. To prove that we will first establish a useful property of a canonical element *s*.

#### Lemma 3

For every canonical element $s \in \mathbb {A}$, node $n \in N$ and element $\mathbf {x}_n \in s(n)$, we have that $\mathsf {ext}(\mathbf {x}_n) \cap \gamma (s) \not = \emptyset$.

Stated another way, the lemma claims that for any canonical *s*, any $\mathbf {x}_n \in s(n)$ can be extended to form some element of $\gamma (s)$. A proof is provided in the extended version.

#### Theorem 2

An element *s* is canonical (Definition 10) if and only if $s = \alpha (S)$ for some concrete set *S*.

Ideally, in abstract interpretation, we would like to work with abstract domain objects that satisfy $s = \alpha (\gamma (s))$. One way to ensure that is to take any given domain element $s_0$ and simply calculate out $\alpha (\gamma (s_0))$ by applying the maps. However, $\gamma (s_0)$ in our domain takes lower dimensional projections and reconstructs a set in the full states pace. It may thus be too expensive to compute. Fortunately, canonical objects satisfy the equality $s = \alpha (\gamma (s))$. Therefore, given any object $s \in \mathbb {A}$ that is not necessarily canonical, we would like to make it canonical: I.e, we seek an object $\hat{s}$ such that $\gamma (\hat{s}) = \gamma (s)$, but $\hat{s}$ is canonical. As mentioned earlier, directly computing $\hat{s} = \alpha (\gamma (s))$ can be prohibitively expensive, depending on the domain. We now describe a *message passing* approach.

First, we convert the tree *T* to a rooted tree by designating an arbitrary node $r \in N$ as the root of the tree.

**Message Passing along Edges:** Let $(n_1, n_2)$ be an edge of the tree and *s* be an abstract element. A message from $n_1$ to $n_2$ is defined as the set $\mathsf {msg}(s, n_1 \rightarrow n_2):\ \mathsf {proj}(s(n_1), \textsc {CV}(n_1, n_2))$. In other words, we project the set $s(n_1)$ along the dimensions that are common to $(n_1, n_2)$.

Once a node $n_2$ receives $M: \mathsf {msg}(s, n_1 \rightarrow n_2)$, it processes the message by updating $s(n_2)$ as $s(n_2) := s(n_2) \cap \mathsf {ext}_{\textsc {verts}(n_2)}(M)$. In other words, it intersects the message (extended to the dimensions in $n_2$) with the current set that is associated with $n_2$.

#### Example 7

Consider a tree decomposition with three nodes $\{n_1, n_2, n_3 \}$ and the edges $(n_1, n_2)$ and $(n_2, n_3)$. Let $\textsc {verts}(n_1): \{ x_1, x_2 \}$, $\textsc {verts}(n_2): \{ x_2, x_4\}$ and $\textsc {verts}(n_3):\ \{x_2, x_3\}$. Let *D* be the domain $\{1,2,3,4\}^4$. Consider the abstract element *s*:A message $\mathsf {msg}(s, n_1 \rightarrow n_2)$ is given by the set . This results in the new abstract object $s'$ wherein the element  is removed from $s(n_2)$:


**Upwards Message Passing:** The upwards message passing works from leaves up to the root of the tree according to the following two rules:

1. First, each leaf of the tree *n* passes a message to its parent $n_p$. The parent node $n_p$ intersects its current value $s(n_p)$ with the message to update its current set.
2. After a node has received (and processed) a message from all its children, it passes a message up to its parent, if one exists.

The upwards message passing terminates at the root since it does not have a parent to send a message to.

#### Example 8

Going back to Example 7, we designate $n_2$ as the root and the upwards pass sends the messages $\mathsf {msg}(s, n_1 \rightarrow n_2)$ and $\mathsf {msg}(s, n_3 \rightarrow n_2)$. This results in the following updated element:


**Downwards Message Passing:** The downwards message passing works from the root down to the leaves.

1. To initialize, the root sends a message to all its children.
2. After a node has received (and processed) a message from its parent, it sends a message to all its children.

The overall procedure to make a given abstract object *s* canonical is as follows: (a) perform an upwards message passing phase and (b) perform a downwards message passing phase.

#### Example 9

Going back to Example 8, the downward message passing phase sends messages from $n_2 \rightarrow n_1$ and $n_2 \rightarrow n_3$. The resulting element $\hat{s}$ isOn the other hand, it is important to perform message passing upwards first and then downwards second. Reversing this does not yield a canonical element. For instance going back to Example 7, if we first performed a downwards pass from $n_2$, the result is unchanged:Performing an upwards pass now yields the element $s_2$:However this is not canonical, since the element  in $s_2(n_1)$ violates the requirement over the edge $(n_1, n_2)$.

Let $\hat{s}$ be the resulting abstract object after the message passing procedure finishes.

#### Theorem 3

The result of message passing $\hat{s}$ is a canonical object, and it satisfies $\gamma (\hat{s})= \gamma (s)$.

#### Proof (Sketch)

First, we note that whenever a message is passed for an abstract value *s* from node *m* to *n* along an edge (*m*, *n*) resulting in a new abstract value $s'$: **(P1)** $\gamma (s') = \gamma (s)$; and **(P2)** the projection of $s'(n)$ along the dimensions $\textsc {CV}(m,n)$ is now contained in that of $s'(m)$ along $\textsc {CV}(m,n)$. Furthermore, property **(P2)** remains unchanged regardless of any future messages that are passed along the tree edges.

Next, it is shown that after each upwards pass, when a message is passed, property **(P2)** (stated above) holds for each node *m* and its parent node *n* since a message is passed from *m* to *n*. During the downwards pass, property **(P2)** holds for each node *n* and its child node *m* in the tree. Combining the two, we note that for each edge (*m*, *n*) in the tree, we have property **(P2)** in either direction guaranteeing that $\mathsf {proj}(s^*(m), \textsc {CV}(m,n))= \mathsf {proj}(s^*(n), \textsc {CV}(m,n))$, for the final result $s^*$, or in other words that $s^*$ is canonical.

### Decomposable Sets and Post-conditions

We have already noted that for any concrete set over $S \subseteq D$, the process of abstracting it by projecting into nodes of a tree *T*, and re-concretizing it is “lossy”: I.e, $S \subseteq \gamma (\alpha (S))$. In this section, we study “tree decomposable” concrete sets *S* for which $\gamma (\alpha (S)) = S$. Ideally, we would like to prove that if a set *S* is tree decomposable then so is the set $\mathsf {post}(S, \varPi )$ of next states. However, we will disprove this by showing a counterexample. Nevertheless, we will present an analysis of why this fact fails and suggest approaches that can “manage” this loss in precision.

#### Definition 11 (Decomposable Sets)

We say that a set *S* is tree decomposable given a tree *T* iff $\gamma (\alpha (S)) = S$.

This is in fact a “global” definition of decomposability. In fact, a nice “local” definition can be provided that is reminiscent of the notion of conditional independence in graphical models. We will defer this discussion to an extended version of this paper due to space limitations.

#### Example 10

Consider set  and tree *T* below: 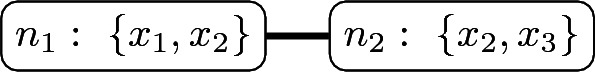 We wish to check if *S* is *T*-decomposable. We have $s:\alpha (S)$ asNow, . We note that the set *S* is not tree decomposable. On the other hand, one can verify that the set  is tree decomposable.

The following lemma will be quite useful.

#### Lemma 4

Let $S_1, S_2$ be tree decomposable sets over *T*. Their intersection is tree decomposable.

Let $\varPi$ be a transition system over system variables in $\mathbf {x}\in D$. For a given set $S \subseteq D$, us define the post-condition $\mathsf {post}(S, \varPi )$ to be the set of states reachable in one step starting from some state in *S*:

$$\begin{aligned} \mathsf {post}(S, \varPi ):\ \{ \mathbf {x}'\ |\ \mathbf {x}\in S,\ \mathbf {x}' = \mathsf {\mathsf {eval}}(f, \mathbf {x})\} \,. \end{aligned}$$

Let us also consider a transition relation *R* over pairs of states $(\mathbf {x}, \mathbf {x}') \in D \otimes D$:

$$\begin{aligned} R = \{ (\mathbf {x}, \mathbf {x}')\ |\ \mathbf {x}, \mathbf {x}'\in D\ \text{ and }\ \mathbf {x}' = \mathsf {\mathsf {eval}}(f, \mathbf {x}) \} \,. \end{aligned}$$

The relation *R* can be viewed as the intersection of *n* relations: $R:\ \bigcap _{x_j \in X} R_j$, wherein

$$\begin{aligned} R_j:\ \{ (\mathbf {x}, \mathbf {x}')\ |\ \mathbf {x}, \mathbf {x}'\in D\ \text{ and }\ \mathbf {x}_j' = \mathsf {eval}(f_j, \mathbf {x}) \} \,. \end{aligned}$$

In other words, $R_j$ is a component of *R* that models the update of the system variable $x_j$. Also for each $x_j \in X$, let $e_j:\ \mathsf {inps}(f_j) \cup x_j$ be the inputs to the update function $f_j$ and the node $x_j$ itself.

Given the tree *T*, we define the extended tree $T'$ as having the same node set *N* and edge set *C* as *T*. However, $\textsc {verts}_{T'}(n) = \textsc {verts}_T(n) \cup \{ x_j'\ | x_j \in \textsc {verts}_T(n) \}$. Note that $T'$ with the labeling $\textsc {verts}_{T'}$ satisfies all the condition of a tree decomposition for a graph *G* save the addition of vertices $x_i'$ in each node of the tree. We will write $\textsc {verts}'(n)$ to denote the set $\textsc {verts}_{T'}(n)$.

#### Lemma 5

The transition relation *R* of a system $\varPi$ is tree $T'$ decomposable.

The proof is provided in the extended version and is done by writing *R* as an intersection of tree decomposable relations $R_j$, and appealing to Lemma 4.

First, we show the negative result that the image of a tree (*T*) decomposable set under a tree ($T'$) decomposable transition relation is not tree decomposable, in general.

#### Example 11

Let $X = \{ x_1, x_2, x_3\}$ and consider again the tree decomposition from Example 10. Let *S* be the set , wherein we use the wild card character as notation that can be substituted for any element in the set $\{1, 2\}$. Therefore, we take *S* to be a set with 8 elements. Clearly *S* is tree decomposable in the tree *T* from Example 10.

Consider the transition relation *R* that will be written as the intersection of three transition relations:

$$\begin{aligned} R_1: \{ (X, X')\ |\ x_1' = x_2 \},\ R_2: \{ (X, X')\ |\ x_2' \in \{1,2\} \},\ R_3' :\ \{ (X, X')\ |\ x_3' = x_2 \} \,. \end{aligned}$$

Clearly *R* is tree $T'$ decomposable. We can now compute the post-condition of *S* under this relation. The reader can verify the post-condition . However, $\hat{S}$ is not tree decomposable. We note that $\hat{s}:\ \alpha (\hat{S})$ is the set  and . Therefore $\gamma (\hat{s})$ is the set .

As noted above, the set *R* is tree $T'$ decomposable. If *S* is tree decomposable, we can extend *S* to a set $S':\ \mathsf {ext}_{X'}(S)$ that is now defined over $X \cup X'$ and is also tree decomposable. As a result $S'\cap R$ is also tree decomposable. However, the postcondition of *S* is the set $\mathsf {proj}(S' \cap R , X')$. Thus, the key operation that failed was the projection operation involved in computing the post-condition. This suggests a possible solution to this issue albeit an expensive one: at each step, we maintain the reachable states using both current and next state variables, thus avoiding projection. In effect, the reachable states at the $i^{th}$ step will be entire trajectories of the system expressed over variables $X_0 \cup X_1 \cup \cdots X_i$. This is clearly not practical. However, a more efficient solution is to note that some of the current state variables can be projected out without losing the tree decomposability property. Going back to Example 11, we note that we can safely project away $\{x_1, x_3\}$, while maintaining the new reachable set in terms of $(x_2, x_1', x_2', x_3')$. In this way, we may recover the lost precision back.

In conclusion, we note that tree decompositions may lose precision over post-conditions. However, the loss in precision can be avoided if carefully selected “previous state variables” are maintained as the computation proceeds. The question of how to optimally maintain this information will be investigated in the future.

## Grid-Based Interval Analysis

We now combine the ideas to create a disjunctive interval analysis using tree decompositions. The main idea here is to apply tree decompositions not to the concrete set of states but to an abstraction of the concrete domain by grid-based intervals.

We will now describe the interval-based abstraction of sets of states dynamical system $\varPi$ in order to perform over-approximate reachability analysis. Let us fix a system $\varPi : \left\langle \mathbf {x}, \mathbf {w}, D, W, f, X_0, U \right\rangle$ as defined in Definition 1. We will assume that the domain of state variables *D* is a hyper-rectangle given by $D: [L(x_1), U(x_1)] \times \cdots \times [L(x_n), U(x_n)]$ for $L(x_j), U(x_j) \in \mathbb {R}$ and $L(x_j) \le U(x_j)$ for each $j = 1, \ldots , n$. In other words, each system variable $x_j$ lies inside the interval $[L(x_j), U(x_j)]$. Likewise, we will assume that $W: \prod _{k=1}^m [L(w_k), U(w_k)]$ such that $L(w_k) \le U(w_k)$ and $L(w_k), U(w_k) \in \mathbb {R}$.

We will consider a *uniform* cell decomposition wherein each dimension is divided into some natural number $M\,{>}\,0$ of equal sized subintervals. The $i^{th}$ *subinterval* of variable $x_j$ is denoted as $\mathsf {subInt}(x_j, i)$, and is given by $[L(x_j) + i \delta _j, L(x_j) + (i+1) \delta _j]$ for $i = 0, \ldots , M-1$ and $\delta _j: \frac{(U(x_j) - L(x_j))}{M}$. Similarly, we will define $\mathsf {subInt}(w_k, i)$ for disturbance variables $w_k$ whose domains are also divided into *M* subdivisions. The overall domain $D \times \mathcal {W}$ is therefore divided into $M^{m+n}$ cells wherein each cell is indexed by a tuple of natural numbers $\mathbf {i}: \left\langle i_1, \ldots , i_n, i_{n+1}, \ldots , i_{n+m} \right\rangle$, such that $i_j \in \{0, \ldots , M-1\}$ and the cell corresponding to $\mathbf {i}$ is given by:

1 $$\begin{aligned} \gamma _C(\mathbf {i}) : \ \prod _{j=1}^n \mathsf {subInt}(x_j, \mathbf {i}_j)\ \times \ \prod _{k=1}^m \mathsf {subInt}(w_k, \mathbf {i}_{n+k}) \end{aligned}$$

### Definition 12 (Grid-Based Abstract Domain)

The grid based abstract domain is defined by the set $\mathcal {C}:\ \mathcal {P}(\mathbf {i} \in \{ 0,\ldots , M\}^{m+n})$, wherein each abstract domain element is a set of grid cells. The sets are ordered simply by set inclusion $\subseteq$ between sets of grid cells. The abstraction map $\alpha _C: \mathcal {P}(D) \rightarrow \mathcal {C}$ is defined as follows:

$$\begin{aligned} \alpha _C(S): \{ \mathbf {i} \in \mathcal {C}\ | \ \gamma _C(\mathbf {i}) \cap S \not = \emptyset \} \,. \end{aligned}$$

The concretization map $\gamma _C$ is defined above in (1).

### Definition 13 (Interval Propagator)

An interval propagator (IP) is a higher order function that takes in the description of a function *f* with *k* real-valued inputs and *p* real valued outputs, and an interval $I: [l_1, u_1] \times \cdots \times [l_k, u_k]$ and outputs an interval (hyperrectangle over $\mathbb {R}^p$) $\textsc {IntvlProp}(f, I)$ such that the following soundness guarantees hold:

$$\begin{aligned} (\forall \mathbf {x}\in D) \bigwedge _{j=1}^k \mathbf {x}_j \in [l_j, u_j] \ \Rightarrow \ \mathsf {eval}(f, \mathbf {x}) \in \textsc {IntvlProp}(f,I) \,. \end{aligned}$$

In practice, interval arithmetic approaches have been used to build sound interval propagators [33]. However, they suffer from issues such as the *wrapping effect* that make their outputs too conservative. This can be remedied by either (a) performing a finer subdivision of the inputs (i.e, increasing *M*) to ensure that the intervals *I* being input into the $\textsc {IntvlProp}$ are sufficiently small to guarantee tight error bounds; or (b) using higher order arithmetics such as affine arithmetic or Taylor polynomial arithmetic [25, 32].

The interval propagator serves to define an abstract post-condition operation over sets of cells $\hat{S} \subseteq \mathcal {C}$. Given such a set, $\hat{S}$, we compute the post condition in the abstract domain. Informally, the post condition is given (a) by iterating over each cell in *S*; and (b) computing the possible next cells using $\textsc {IntvlProp}$. Formally, we define the abstract post operation as follows:

$$\begin{aligned} \mathsf {post}_C(\hat{S}, \varPi ):\ \bigcup _{\mathbf {i} \in \hat{S}} \alpha _C( \textsc {IntvlProp}(f, \gamma _C(\mathbf {i})) ) \,. \end{aligned}$$

Given this machinery, an abstract *T*-step reachability analysis is performed in the standard manner: (a) abstract the initial state; (b) compute post condition for *T* steps; and (c) check for intersections of the abstract states with the abstraction of the unsafe set. We can also define and use widening operators to make the sequence of iterates converge. The grid based abstract domain can offer some guarantees with respect to the quality of the abstraction. For instance, we can easily bound the Hausdorff distance between the underlying concrete set and the abstraction as a function of the discretization sizes $\delta _j$. However, the desirable properties come at a high computational cost since the number of cells grows exponentially in the number of system and disturbance variables.

### Tree Decomposed Analysis

We now consider a tree-decomposed approach based on the concept of nodal abstractions. The key idea here is to perform the grid-based abstraction not on the full set of system and disturbance variables, but instead on individual *nodal* abstractions over a tree decomposition *T*.

#### Definition 14 (Nodal Abstractions)

 A nodal abstraction $\textsc {Nodal}$ $\textsc {Abstraction}(\varPi , n)$ corresponding to a node $n \in N$ is defined as follows

1. The set of system variables are given by $X_n:\ \textsc {verts}_X(n)$ with domain given by $D_n:\ \mathsf {proj}(D, X_n)$.
2. The initial states are given by $\mathsf {proj}(X_0, X_n)$.
3. The unsafe set is given by $\mathsf {proj}(U, X_n)$.
4. The set of disturbance variables are $Y_n:\ \textsc {verts}_W(n)$ with domain given by $W_n:\ \mathsf {proj}(W, W_n)$.
5. The updates are described by a *relation* $R(X_n, X_n')$ that relate the possible current states $X_n$ and next states $X_n'$. The relation is constructed as a conjunction of assertions over variables $x_i, x_i'$ wherein $x_i \in X_n$.
  - If the update $x_i := f_i(\mathbf {x}, \mathbf {w})$ is associated with the node *n*, we add the conjunct $x_i' = f_i(X_n, W_n)$, noting that the proper inputs to $f_i$ are contained in $\textsc {verts}(n)$.
  - Otherwise, $x_i' \in \mathsf {proj}(D, \{x_i\})$ that simply states that the next state value of the variable $x_i$ is some value in its domain.

Given a system $\varPi$, the nodal abstraction is a conservative abstraction, and therefore, it preserves reachability properties.

#### Lemma 6

For any reachable state $\mathbf {x}$ of $\varPi$ at time *t*, its projection $\mathsf {proj}(\mathbf {x}, X_n)$ is a reachable state of $\textsc {NodalAbstraction}(\varPi , n)$ at time *t*.

Since each nodal abstraction involves at most $\omega + 1$ variables, the abstraction at each node can involve at most $M^{\omega +1}$ cells where $\omega$ is the tree width. Also, note that a tree decomposition can be found with tree width $\omega$ that has at most $|X|+|W|$ nodes. This implies that the number of nodal abstractions can be bounded by $(|X|+|W|)$.

Let $\varPi (n): \textsc {NodalAbstraction}(\varPi , n)$ be the nodal abstraction for tree node $n \in N$. For each node $n \in N$, we instantiate a grid based abstract domain for $\varPi (n)$ ranging over the variables $\textsc {verts}_X(n)$. At the $i^{th}$ step of the reachability analysis, we maintain a map $s_i$ each node *n* to a set of grid cells $s_i(n)$ defined over $\textsc {verts}(n)$.

1. Compute $$\hat{s_i}(n): \mathsf {post}_C(s_i(n), \varPi (n))$$.
2. Make $\hat{s_i}$ canonical using message passing between nodes to obtain $s_{i+1}$.

The message passing is performed not over projections of concrete states but over cells belonging to the grid based abstract domain. Nevertheless, we can easily extend the soundness guarantees in Theorem 3 to conclude soundness of the composition.

Once again, we can stop this process after *T* steps or use widening to force convergence. We now remark on a few technicalities that arise due to the way the tree decomposition is constructed.

**Intersections with Unsafe Sets:** Checking for a non-empty intersection with the unsafe sets may require constructing concrete cells over the full dimensional space if the unsafe sets are not tree decomposable for the tree *T*. However in many cases, the unsafe states are specified as intervals over individual variables, which yields a tree decomposable set. In such cases, we need to intersect the abstraction at each node with the unsafe set and perform message passing to make it canonical before checking for emptiness.

**Handling Guards and Invariants:** We have not discussed guards and invariants. It is assumed that such guards and invariants are tree decomposable over the tree *T*. In this case, we can check which abstract cells have a non-empty intersection with the guard using message passing. The handling of transition systems with guards and invariants will be discussed as part of future extensions.

## Experimental Evaluation

In this section, we describe an experimental evaluation of our approach over a set of benchmark problems. Our evaluation is based on a C++-based prototype implementation that can read in the description of a nonlinear dynamical system over a set of system and disturbance variables. The dynamics can currently include polynomials, rational functions and trigonometric functions. Our implementation uses the MPFI library to perform interval arithmetic over the grid cells [36]. We use the HTD library to compute tree decompositions [1]. The system then computes a time-bounded reachable set over the first *T* steps of the system’s execution. Currently, we plot the results and compare the reachable set estimates against simulation data. We also compare the reachable sets computed by the tree decomposition approach against an approach without using tree decompositions. However, we note that the latter approach timed out on systems beyond 4 state variables.

**Table 1.** Results on benchmark examples. |*X*|: Number of state variables, |*W*|: number of disturbance variables, Tree Decomp.: reachability using tree decompositions, Monolithic: reachability analysis without tree decompositions. SAPO: number of directions (|*L*|), number of bundles (|*T*|) and running time. All timings are reported in seconds on a Macbook pro laptop running MacOS 10.14 with 16 GB RAM and 3.4 GHz Intel core i7 processor. Reachability analysis was carried out for 15 time steps.

Name	|*X*|	|*W*|	Tree Width	Tree Decomp.		Monolithic		SAPO	
				Time	# Cells	Time	# Cells	(|*L*|, |*T*|)	Time
System # 1	3	1	1	14.4	0.22M	1047.6	7.6M	-n/a-	
System # 2	4	1	2	2.7	24K	652	3.1M	-n/a-	
SIR [23, 40]	3	0	1	4.1	95K	143	2M	(3,1)	0.1
1D-Lattice-10 [39]	10	0	2	99	1.1M	TO (1.5 h)		(16,6)	679
Ebola-epidemic [14]	5	0	2	799.4	1.9M	TO (1.5 h)		(5,5)	0.02
p53-gene-reg [31]	6	0	2	135.8	98K	TO (1.5 h)		-n/a-	
Influenza-epidemic [22]	4	0	2	517.9	1.4M	TO (1.5 h)		(7,4)	0.1
Coupled-vanderpol	6	0	2	10.5	0.1M	TO (1.5 h)		(10,5)	2.5
Laub-Loomis [20, 30]	7	0	3	1755.1	2.6M	TO (1.5 h)		(12,6)	1.8
Honeybee* [9, 23]	6	4	3	206.1	2.1M	TO (1.5 h)		(8,4)	0.7
Phosporelay [22]	7	0	3	1566.2	7.5M	TO (1.5 h)		(10,4)	1.2
Coord. Vehicles (1)	5	1	2	150.2	0.5M	TO (1.5 h)		-n/a-	
Coord. Vehicles (2)	10	2	2	1175.2	2M	TO (1.5 h)		-n/a-	
Coord. Vehicles (4)	20	4	2	2206.7	3.9M	TO (1.5 h)		-n/a-	

Table 1 presents the results over a small set of challenging nonlinear systems benchmarks along with a comparison to two other approaches (a) the approach without tree decomposition and (b) the tool SAPO [22] which computes time bounded reachable sets for polynomial systems using the technique of parallelotope bundles described by Dreossi et al. [23]. The benchmarks range in number of system variables from 3 to 20 state variables. We describe the sources for each benchmark where appropriate. Note that the SAPO tool does not handle nonpolynomial dynamics or time varying disturbances at the time of writing.

The treewidths range from 1 for the simplest system (Example 1) to 3 for the 7-state Laub Loomis oscillator example [30]. We note that the tree decomposition was constructed within 0.01 s for all the examples. We also note that systems with as many as 20 state variables are handled by our approach whereas the monolithic approach cannot handle systems beyond 4 state variables. We now compare the results of our approach to that of the monolithic approach on the two cases where the latter approach completed.

**System # 1:** Consider again the system from Example 1 with 3 state variables and 1 disturbance. We have already noted a tree decomposition of tree width 1 for this example.

**Fig. 1.**
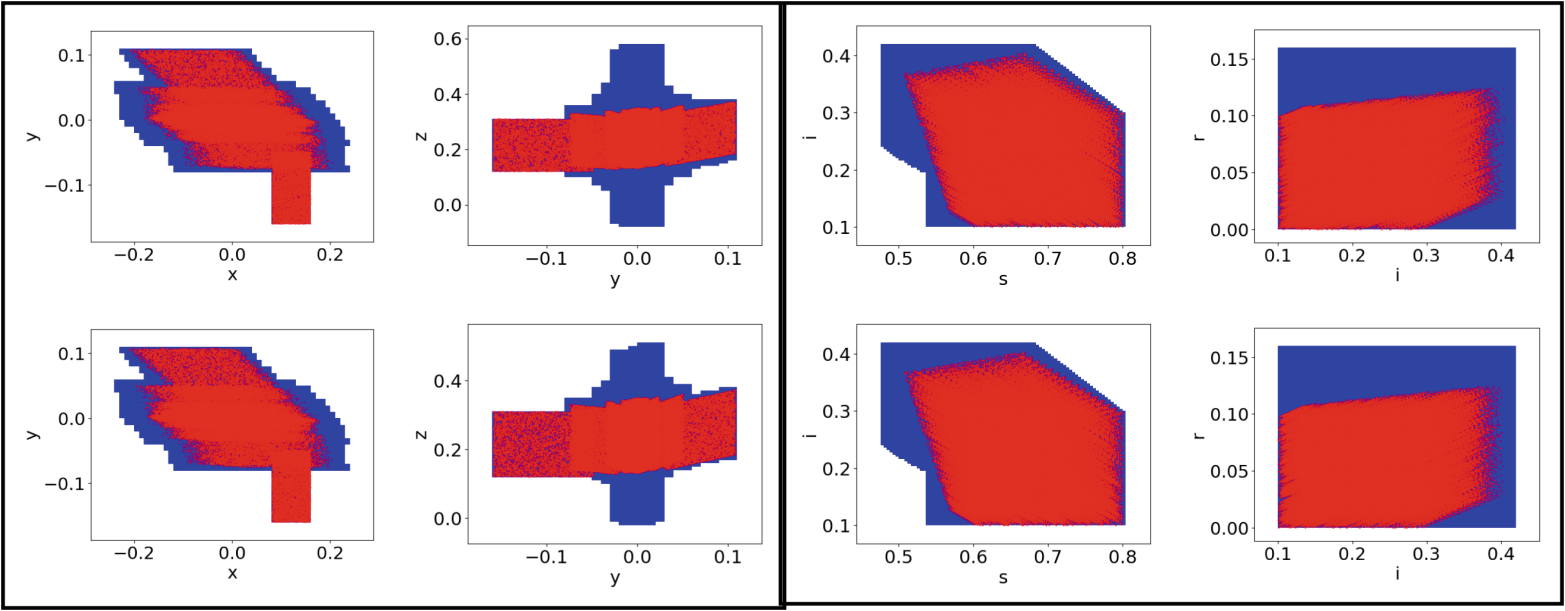
Reachable set projections (shaded blue) for System# 2 (left) and the SIR model [22] (right). Top: tree decomposition approach and Bottom: monolithic approach without tree decompositions. Reachable sets are identical for the SIR model. Note the difference in range of *z* for the system #2. The red dots show the results of simulations. (Color figure online)

**System # 2:** In this example, we consider a system over 4 state variables $\{x,y,z, w\}$ and one disturbance variable $w_1$.The domains include $(x,y,z,w) \in [-1,1]^4$ and divided into $16\times 10^{8}$ grid cells (200 for each state variable). The disturbance $w_1 \in [-0.1,0.1]$. The initial conditions are $x \in [0.08, 0.16], y\in [-0.16, -.05], z \in [0.12, 0,31]$ and $w \in [-0.15, -0.1]$. We obtain a tree decomposition of width 2, wherein the nodes include $n_1: \{ x, y, w_1\}$, $n_2: \{ y, z\}$ and $n_3:\ \{ x, w\}$ with the edges $(n_1, n_2)$ and $(n_1, n_3)$.

Figure 1 compares the resulting reachable sets for the tree decomposed reachability analysis versus the monolithic approach. We note differences between the two reachable sets but the loss in precision is not significant.

**Coordinated Vehicles:** In this example, we study nonlinear vehicle models of vehicles executing coordinated turns. Each vehicle has states $(x_i, y_i, v_{x,i}, v_{y,i}, \omega )$, representing positions, velocities and the rate of change in the yaw angle, respectively, with a disturbance . The dynamics are given byThe vehicles are loosely coupled with $\omega _i$ representing the turn rate of the $i^{th}$ vehicle and $\omega _0$ that of the “lead” vehicle. The $i^{th}$ vehicle tries to gradually align its turn rate to that of the lead vehicle. This model represents a simple scenario of loosely coupled systems that interact using a small set of state variables. Applications including models of cardiac cells that are also loosely coupled through shared action potentials [26]. The variables $x_i, y_i$ are set in the domain $[-15,15]$ and subdivided into 300 parts along each dimension. Similarly, the velocities range over $[-10,10]$ and are subdivided into 500 parts each and the yaw rate ranges over $[-0.2,0.2]$ radians/sec and subdivided into 25 parts. The disturbance ranges over $[-0.1,0.1]$. Table 1 reports results from models involving 1, 2 and 4 vehicles. Since they are loosely coupled, the treewidth of these models is 2.

**Laub-Loomis Model:** The Laub-Loomis model is a molecular network that produces spontaneous oscillations for certain values of the model parameters. The model’s description was taken from Dang et al. [20]. The system has 7 state variables each of which was subdivided into 100 cells yielding a large state space with $10^{14}$ cells. We note that the tree width of the graph is 3, yielding nodes with upto 4 variables in them.

**Fig. 2.**
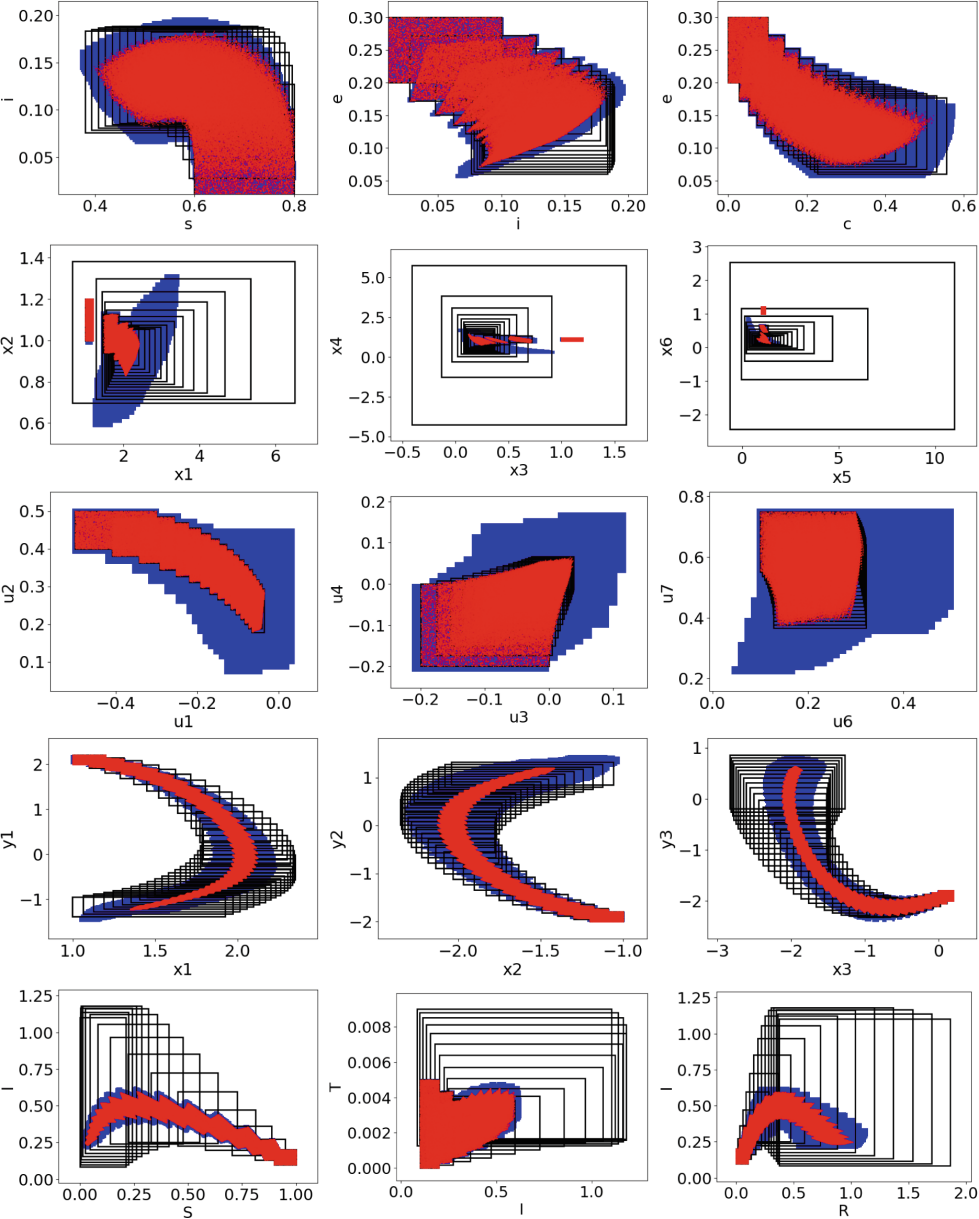
Comparison of various projections of the reachable sets computed by our approach shown in blue, the reachable set computed by SAPO shown as black rectangles and states obtained through random simulation shown in red dots. Top row: ebola model, second row: phosporelay, third row: 1d-lattice-10, fourth row: vanderpol (35 steps) and bottom row: influenza model. (Color figure online)

**Comparison with SAPO.** SAPO is a state-of-the-art tool that uses polytope bundles and Bernstein polynomials to represent and propagate reachable sets for polynomial dynamical systems [22, 23]. We compare our approach directly on SAPO for identical models and initial sets. Note that SAPO does not currently handle non-polynomial models or models with time-varying disturbances. Table 1 shows that SAPO is orders of magnitude faster on all the models, with the sole exception of the 1D-Lattice-10 model. Figure 2 shows the comparison of the reachable sets computed by our approach (shaded blue region) against those computed by SAPO (black rectangles) for five different models. We note that for three of the models compared, neither reachable set is contained in the other. For the one dimensional lattice model, SAPO produces a better reachable set, whereas our approach is better for the influenza model. We also note that both for our approach the precision can be improved markedly by increasing the number of subdivisions, albeit at a large computational cost that depends on the treewidth of the model. The same is true for SAPO, where the number of directions and the template sizes have a non-trivial impact on running time.

**Models with Large Treewidths.** We briefly report on a few models that we attempted with large treewidths. For such models, our approach of decomposing the space into cells becomes infeasible due to the curse of dimensionality.

A model of how honeybees select between different sites [9, 23] has 6 variables and its tree width is 5 with a single tree node containing all state variables. However, the large treewidth is due to two terms in the model which are replaced by disturbance variables that overapproximate their value. This brings down the treewidth to 3, making it tractable for our approach. Details of this transformation are discussed in our extended version. Treewidth reduction using abstractions is an interesting topic for future work.

We originally proposed to analyze a 2D grid lattice model taken from Vleck et al [39]. However, a 2D $10 \times 10$ lattice model has a dependency hypergraph that forms a $10\times 10$ grid with treewidth 10. Likewise, the 17-state crazyflie benchmark for SAPO [22] could not be analyzed by our approach since its treewidth is too large.

## Conclusions

We have shown how tree decompositions can define an abstract domain that projects concrete sets along the various subsets of state variables. We showed how message passing can be used to exchange information between these subsets. We analyze the completeness of our approach and show that the abstraction is lossy due to the projection operation. We show that for small tree width models, a gridding-based analysis of nonlinear system can be used whereas such approaches are too expensive when applied in a monolithic fashion. For the future, we plan to study tree decompositions for abstract domains such as disjunctions of polyhedra, parallelotope bundles and Taylor models. The process of model abstraction to reduce treewidth is another interesting future possibility.
